# Role of Matrix Metalloproteinase 13 in Both Endochondral and Intramembranous Ossification during Skeletal Regeneration

**DOI:** 10.1371/journal.pone.0001150

**Published:** 2007-11-07

**Authors:** Danielle J. Behonick, Zhiqing Xing, Shirley Lieu, Jenni M. Buckley, Jeffrey C. Lotz, Ralph S. Marcucio, Zena Werb, Theodore Miclau, Céline Colnot

**Affiliations:** 1 Department of Anatomy and Biomedical Sciences Graduate Program, University of California at San Francisco, San Francisco, California, United States of America; 2 Cellular and Molecular Biology Laboratory, Department of Orthopaedic Surgery, University of California at San Francisco, San Francisco General Hospital, San Francisco, California, United States of America; 3 Orthopaedic Bioengineering Laboratory, Department of Orthopaedic Surgery, University of California at San Francisco, San Francisco, California, United States of America; University of California at Berkeley, United States of America

## Abstract

Extracellular matrix (ECM) remodeling is important during bone development and repair. Because matrix metalloproteinase 13 (MMP13, collagenase-3) plays a role in long bone development, we have examined its role during adult skeletal repair. In this study we find that MMP13 is expressed by hypertrophic chondrocytes and osteoblasts in the fracture callus. We demonstrate that MMP13 is required for proper resorption of hypertrophic cartilage and for normal bone remodeling during non-stabilized fracture healing, which occurs via endochondral ossification. However, no difference in callus strength was detected in the absence of MMP13. Transplant of wild-type bone marrow, which reconstitutes cells only of the hematopoietic lineage, did not rescue the endochondral repair defect, indicating that impaired healing in *Mmp13^−/−^* mice is intrinsic to cartilage and bone. *Mmp13^−/−^* mice also exhibited altered bone remodeling during healing of stabilized fractures and cortical defects via intramembranous ossification. This indicates that the bone phenotype occurs independently from the cartilage phenotype. Taken together, our findings demonstrate that MMP13 is involved in normal remodeling of bone and cartilage during adult skeletal repair, and that MMP13 may act directly in the initial stages of ECM degradation in these tissues prior to invasion of blood vessels and osteoclasts.

## Introduction

Bone is noteworthy in that it does not heal through the formation of scar tissue, but rather through a regenerative process that resembles fetal skeletal development. During bone healing much of the initial developmental program is conserved, from the various cell types involved to the genetic mechanisms regulating cell differentiation [1]–[3]. As in development, repair of long bones by endochondral ossification begins with the production of cartilage at the injury site. The chondrocytes of the fracture callus deposit an extracellular matrix (ECM) comprised of type II collagen (Col2) and aggrecan, then differentiate into hypertrophic chondrocytes that deposit an ECM comprised of type X collagen (Col10). This ECM is then partially mineralized, resorbed and replaced by a matrix comprised predominantly of type I collagen (Col1). This process is regulated by the action of invading osteoblasts and osteoclasts. These cells, whose development and function are intimately linked, continue to remodel this regenerated tissue into mature bone until the fracture is consolidated. Other aspects of skeletal repair differ from skeletal development. For example, skeletal repair may be influenced by the mechanical environment. While non-stabilized fractures heal via endochondral ossification, stabilized fractures heal via intramembranous ossification. In this process, mesenchymal precursors recruited to the site of injury differentiate only along the osteoblastic lineage and produce both compact (cortical) and spongy (cancellous) bone in the absence of cartilage production [4], [5].

Skeletal elements are rich in ECM and the remodeling of this ECM is central to both development and repair [3], [5]. Matrix remodeling in both of these processes is regulated by many of the same proteases, and these enzymes may determine the rate and effectiveness of the development and repair programs [6]. The role of matrix metalloproteinases (MMPs) during bone development has been studied extensively [7]–[12]. MMP13 promotes both the resorption of hypertrophic cartilage from the growth plate and the remodeling of newly deposited trabecular bone during long bone development [9], [10]. Moreover, other work has pointed to the requirement for MMPs used in bone development for bone repair [3], [5], [13], [14]. These reports have led us to ask whether MMP13 also participates in skeletal repair. In the present study, we employed several models of repair by both endochondral and intramembranous ossification, including non-stabilized and stabilized fracture models as well as a cortical defect model, to elucidate the role of MMP13 during the various stages of healing.

## Results

### Mmp13 is expressed in the non-stabilized fracture callus

**Figure 1:**
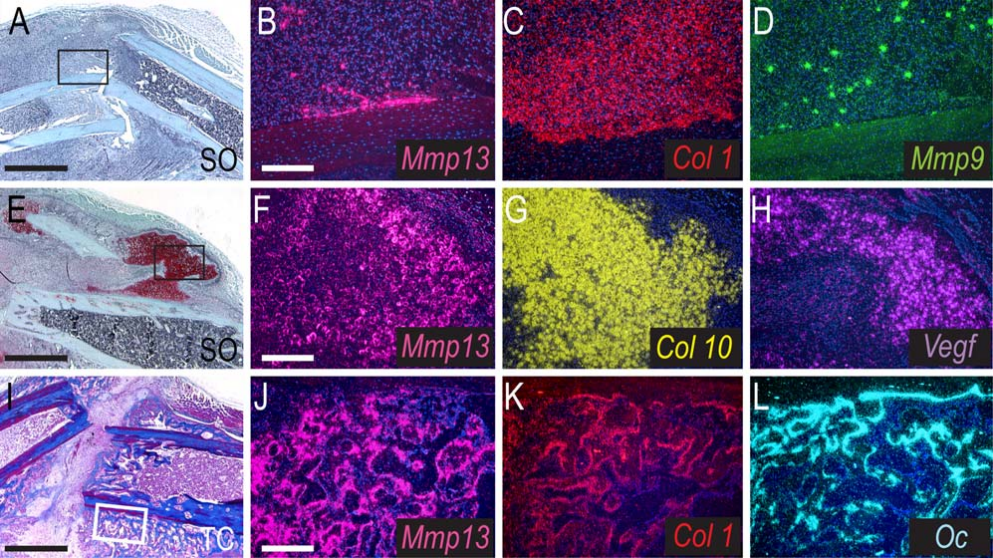
*Mmp13* expression during non-stabilized fracture healing. (Left column) Safranin-O/Fast Green (SO) and Trichrome (TC) stained sagittal sections through the WT callus at 6 (A), 10 (E) and 14 (I) days post-fracture. Cartilage (red) develops during the soft callus phase of healing (A), is resorbed during the hard callus phase (E) and is replaced by bone (blue, I). (Middle/Right column) In situ hybridization analyses of *Mmp13* expression and osteoblast/chondrocyte differentiation markers. (B–D) At 6 days post-fracture, *Mmp13* is expressed in the callus and overlaps with *Col1*-expressing cells (early osteoblasts) but not *Mmp9*-expressing cells (osteoclasts and inflammatory cells). (F–H) At day 10, *Mmp13* mRNA is detected in hypertrophic chondrocytes also expressing *Col10* and *Vegf*. (J–L) At day 14, *Mmp13* is expressed in mature osteoblasts co-expressing *Col1* and/or *Oc*. Scale bars: A, E, I = 1mm; B-D, F-H, J-L-200 µm.

We first established which cells express *Mmp13* during endochondral ossification in non-stabilized fractures. We performed in situ hybridization analyses on histological sections through fracture calluses from wild-type (WT) mice using probes for *Mmp13* and a number of cell type-specific marker genes (Fig. 1). At 3 days post-fracture, we observed *Mmp13* expression in regions of activated periosteum that also expressed *Col1* (data not shown). Likewise, at 6 days post-fracture, we observed *Mmp13*-expressing cells within the *Col1* expression domain in *Osteocalcin* (*Oc*)-negative portions of the periosteum, indicating that *Mmp13* is expressed during the early stages of healing by immature osteoblasts (Fig. 1A–D and data not shown). The *Mmp13* expression pattern differed from that of *Mmp9*, indicating that *Mmp13* was not upregulated in newly recruited inflammatory cells and osteoclasts (Fig. 1B–D) as observed previously (Colnot et al., 2003). At 10 days post-fracture, *Mmp13* expression in the cartilage overlapped with *Col10* and *Vascular endothelial growth factor* (*Vegf*) expression, indicating that *Mmp13* is expressed by hypertrophic and late hypertrophic chondrocytes (Fig. 1E–H). At 14 days post-fracture, *Mmp13* expression colocalized with *Col1* and *Oc* expression, suggesting that by this time point *Mmp13* is expressed in both immature and mature osteoblasts within the callus (Fig. 1I–L). *Mmp13* expression was detected in osteoblasts throughout the remodeling phase of healing and was not associated with osteoclasts (Fig. 1A–D and data not shown). These results demonstrate that the expression pattern of *Mmp13* in both cartilage and bone tissues during fracture healing parallels that seen during development via endochondral ossification [10], [15], [16] and suggest that MMP13 may play a role in both cartilaginous and bony tissues during fracture healing.

### Mmp13^−/−^ mice accumulate cartilage during non-stabilized fracture healing but cartilage differentiation to hypertrophy is normal

**Figure 2:**
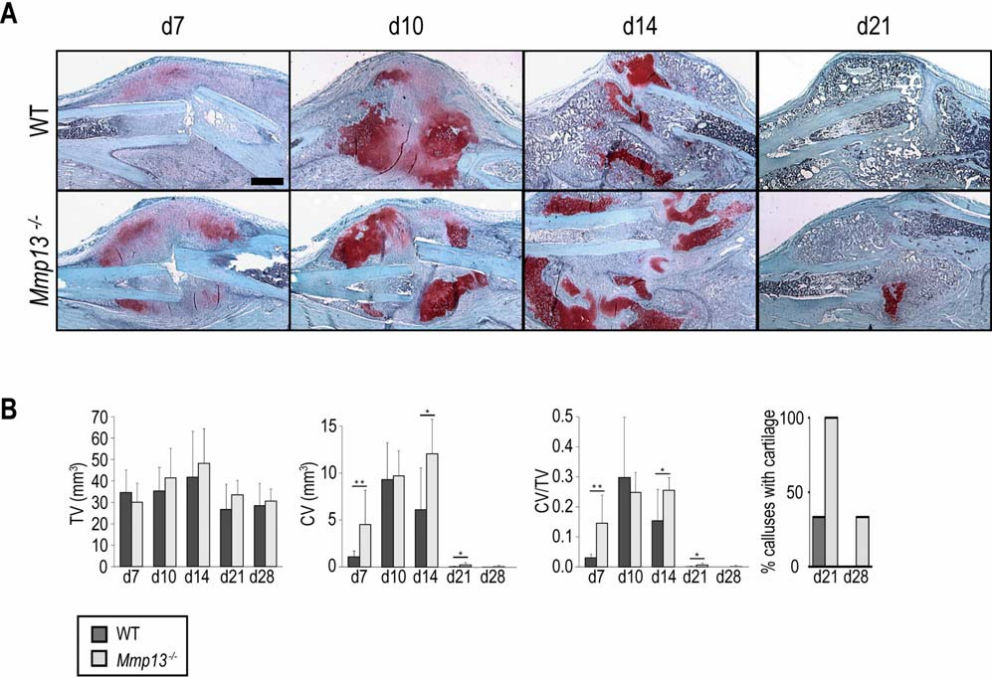
*Mmp13* ^−/−^ mice display an accumulation of cartilage during non-stabilized fracture healing. (A) SO staining of WT and *Mmp13* ^−/− ^fracture callus at 7, 10, 14, and 21 days post-fracture shows that cartilage persists in the *Mmp13* ^−/−^ callus from 14 through 21 days post-fracture. Scale bar = 1 mm (B) Histomorphometric measurements of total callus volume (TV), total cartilage volume (CV) and total cartilage volume as a proportion of total callus volume (CV/TV) in WT and *Mmp13* ^−/−^ mice at day 7 (WT *n* = 6, *Mmp13* ^−/−^ *n* = 6), 10 (WT *n* = 8, *Mmp13* ^−/−^ *n* = 6), 14 (WT *n* = 8, *Mmp13* ^−/−^ *n* = 6), 21 (WT *n* = 6, *Mmp13* ^−/−^ *n* = 6) and 28 (WT *n* = 6, *Mmp13* ^−/−^ *n* = 6). There is a statistically significant increase in total cartilage volume in *Mmp13* ^−/−^ calluses compared with WT at day 7 (**p<0.01), 14 (*p<0.05) and 21 (*p<0.05). There is a statistically significant increase in total cartilage volume as a proportion of total callus volume in *Mmp13* ^−/−^ calluses compared with WT at day 7 (**p<0.01), 14 (*p<0.05) and 21 (*p<0.05). Wilcoxon test, bars represent means ± S.D. At 21 days post-fracture, all *Mmp13^−/−^* calluses contained cartilage as compared to 1/3 of WT. At 28 days post-fracture, only *Mmp13^−/−^* calluses (1/3) still contained cartilage.

Cartilage begins to develop in the non-stabilized fracture callus by days 3 to 7 post-fracture (soft callus phase), peaks at day 10, is being remodeled by day 14 (hard callus phase) and is fully resorbed by day 28 (remodeling phase; [5]). To study the effect of MMP13 deficiency on this process, we created closed, non-stabilized fractures in WT and *Mmp13^−/−^* adult mice and examined the timing and extent of cartilage and bone formation throughout the healing process. Histological analyses indicated that cartilage is formed in *Mmp13^−/−^* calluses, but its remodeling and removal are delayed (Fig 2A). Quantitative analyses confirmed these observations. Histomorphometric analyses revealed that there was no significant difference in total callus volume between WT and *Mmp13^−/−^* samples at any time point examined (Fig. 2B), but there was a significantly greater volume of cartilage and of cartilage as a proportion of total callus volume in *Mmp13^−/−^* calluses at 7, 14 and 21 days post-fracture (Fig. 2B). Examination of the percentage of samples that exhibited cartilage confirmed the delay in cartilage removal in *Mmp13^−/−^* calluses. At day 21, all *Mmp13^−/−^* calluses contained cartilage while only one third of WT calluses did. At day 28, cartilage was still present in one third of *Mmp13^−/−^* calluses but was never observed in WT samples.

**Figure 3:**
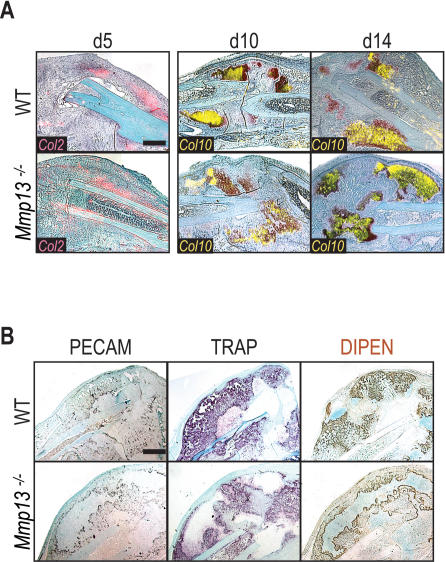
Integral steps in endochondral ossification are unperturbed during non-stabilized fracture repair in *Mmp13^−/−^* mice. (A) Overlay of SO stained sections with in situ hybridization for *Col2* (red) indicate no difference in the early differentiation of chondrocytes in WT and *Mmp13* ^−/−^ calluses at day 5. Overlay of SO stained sections with in situ hybridization for *Col10* (yellow) shows a delay in hypertrophic chondrocyte removal in the *Mmp13* ^−/−^ callus at day 14. Scale bar = 1 mm (B) Cellular analyses of WT and *Mmp13* ^−/−^ calluses at day 14 show that blood vessels (PECAM) and osteoclasts (TRAP) are present in the *Mmp13* ^−/−^ callus while aggrecan cleavage by MMPs (DIPEN epitope) is reduced in the *Mmp13* ^−/−^ callus. Scale bar = 1 mm

To characterize the differences observed during the early stages of healing, we analyzed *Col2* expression in fracture calluses at day 5 by in situ hybridization. We did not detect an alteration in the initial differentiation of chondrocytes in *Mmp13^−/−^* calluses as compared to WT (Fig. 3A), indicating that the difference in cartilage volume observed by day 7 was transient. We next examined chondrocyte hypertrophy in the *Mmp13^−/−^* calluses by investigating *Col10* expression (Fig. 3A). The *Col10*-expression domain was comparable between WT and *Mmp13^−/−^* calluses at days 10 and 14 post-fracture, suggesting that chondrocyte hypertrophy was not altered in the absence of MMP13. These studies demonstrate that the absence of MMP13 did not affect the overall amount of cartilage produced in the callus or its differentiation to hypertrophy, but did affect the removal of hypertrophic cartilage.

To understand the basis for the delayed hypertrophic cartilage removal, we examined angiogenesis and ECM remodeling, two key components of endochondral ossification (Fig. 3B; [5], [10], [12], [17]). Platelet endothelial cell adhesion marker (PECAM) immunostaining (left column) demonstrated that the observed delay in cartilage remodeling/removal was not due to delayed vascular invasion into the *Mmp13* ^−/−^ callus. Staining for Tartrate-resistant acid phosphate (TRAP)-positive cells (Fig. 3B, middle column) demonstrated the delay was also not due to delayed osteoclast recruitment into the *Mmp13* ^−/−^ callus. However, these recruited blood vessels and osteoclasts did not invade the hypertrophic cartilage matrix, suggesting the inability of the cartilage matrix itself to become invaded. We next stained for an epitope of aggrecan, the major proteoglycan of the cartilage ECM [18] which is degraded during the very last stages of chondrocyte differentiation/removal [19]. Cleavage of aggrecan by MMPs, as assessed by immunostaining for the cryptic DIPEN epitope of aggrecan (Fig. 3B, right column; [20]), decreased in the *Mmp13^−/−^* callus indicating that processing of the cartilage ECM in the *Mmp13* ^−/− ^callus was delayed.

### Mmp13^−/−^ mice have increased bone density during non-stabilized fracture healing

**Figure 4:**
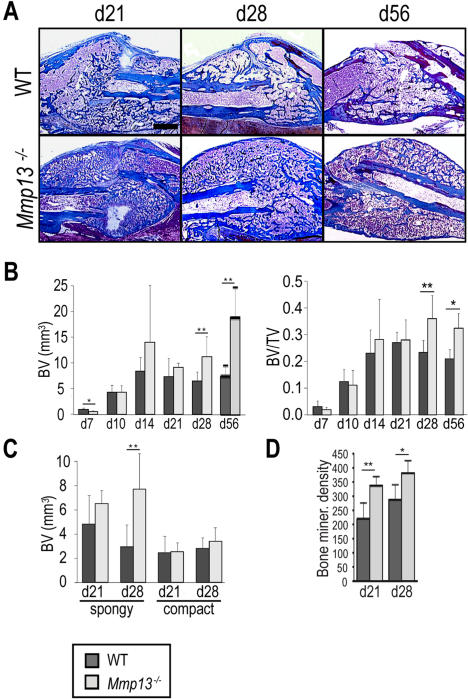
*Mmp13* ^−/−^ mice display increased bone volume during non-stabilized fracture healing. (A) Trichrome staining of WT and *Mmp13* ^−/− ^non-stabilized fracture calluses shows an increase in the amount of bone in the *Mmp13* ^−/−^ callus compared to WT at days 28 and 56 post-fracture. Scale bar = 1 mm (B) Histomorphometric measurements of total bone volume (BV) and total bone volume as a proportion of total callus volume (BV/TV) in WT and *Mmp13* ^−/−^ mice at days 7 (WT *n* = 6, *Mmp13* ^−/−^ *n* = 6), 10 (WT *n* = 8, *Mmp13* ^−/−^ *n* = 6), 14 (WT *n* = 8, *Mmp13* ^−/−^ *n* = 6), 21 (WT *n* = 6, *Mmp13* ^−/−^ *n* = 6), 28 (WT *n* = 6, *Mmp13* ^−/−^ *n* = 6) and 56 (WT *n* = 5, *Mmp13* ^−/−^ *n* = 4) confirm this observation There is a statistically significant decrease in BV in *Mmp13* ^−/−^ calluses compared with WT at day 7 (*p<0.05), but a statistically significant increase in BV and BV/TV in *Mmp13* ^−/−^ calluses compared with WT at days 28 (**p<0.01 and p<0.05 respectively) and 56 (*p<0.05). Wilcoxon test, bars represent means ± S.D. (C) Histomorphometric measurements indicate a statistically significant difference in total spongy bone volume in *Mmp13* ^−/−^ calluses compared with WT at day 28 (**p<0.01) but no difference is detected in total compact bone volume. (D) Micro-CT analyses show that bone mineral density is significantly increased in the *Mmp13^−/−^* callus compared to WT at 21 (**p<0.01) and 28 (*p<0.05) days post-fracture. Bonferroni corrected t-test, bars represent means ± SD.

Since hypertrophic cartilage remodeling is necessary for the replacement of cartilage by bone during healing [5], we asked whether delayed removal of hypertrophic cartilage affects ossification of the fracture callus in *Mmp13^−/−^* mice. Histomorphometric analyses revealed a significantly smaller bone volume in *Mmp13^−/−^* samples at day 7 post-fracture (Fig. 4B), suggesting that the increase in cartilage formation at this early time point may have compromised the initial stages of bone formation. By day 14, however, we did not detect a significantly decreased bone volume nor a significantly decreased bone volume as a proportion of total callus volume in *Mmp13^−/−^* samples compared to WT (Fig. 4B). This suggests that the increased cartilage deposition/decreased cartilage removal observed at this time point does not impair new bone deposition. At later stages of repair, during active remodeling of the new bone in WT calluses, *Mmp13* ^−/−^ calluses had significantly greater callus bone volume and a significantly greater bone volume as a proportion of total callus volume, both at days 28 and 56 post-fracture (Fig. 4B).

By histological examination, we observed that spongy bone accumulated in *Mmp13^−/−^* calluses while the reconstitution of the bone marrow cavity was more advanced in WT calluses (Fig. 4A, day 28, middle column; day 56, right column). Histomorphometric analyses performed on the volumes of new bone in WT and *Mmp13^−/−^* calluses at 21 and 28 days post-fracture confirmed differences in the spongy and compact bone in *Mmp13^−/−^* calluses. At 28 days post-fracture, there was a significantly greater volume of spongy bone in *Mmp13^−/−^* calluses as compared to WT (Fig. 4C). By contrast, there was no significant difference observed in compact bone volume, regardless of timepoint or genotype. Taken together, these results suggest that the increased bone volume observed in the *Mmp13^−/−^* non-stabilized fracture callus resulted from an increase in the spongy bone of the callus.

Furthermore, we performed micro-CT analyses on non-stabilized fracture calluses from WT and *Mmp13^−/−^* mice at 21 and 28 days post-fracture. While no difference in overall callus volume was detected by micro-CT regardless of healing time point or genotype (data not shown), a significant increase in bone mineral density was observed in *Mmp13^−/−^* calluses as compared to WT at 21 and 28 days post-fracture (Fig. 4D). Given that histomorphometric analyses indicated no significant increase in bone volume at 21 days, these results suggest that the bone matrix was not fully mineralized in *Mmp13^−/−^* calluses at this time point. By 28 days, the micro-CT results correlated with that of histomorphometry confirming that MMP13 is required for proper bone remodeling in the non-stabilized fracture callus.

Since the increase in *Mmp13^−/−^* callus bone volume observed by histomorphometry persisted through 8 weeks post-fracture, these results reflected a prolonged change in the bone volume of the mutant callus. We considered the possibility that the impact of MMP13 deficiency on bone volume and bone mineral density in the non-stabilized fracture callus could affect the mechanical properties of these calluses. To test this, we performed mechanical analyses on non-stabilized fracture calluses from WT and *Mmp13^−/−^* mice at 14, 21 and 28 days post-fracture (Table 1). We observed no significant impact on the maximum force at failure of fracture calluses regardless of healing timepoint or genotype (Table 1). Therefore, increased bone volume within the spongy bone compartment of the callus did not change the overall mechanical properties of the tissue.

**Table 1:** Mechanical properties of intact tibiae and unstabilized fracture calluses.

	Healing time (days)			
	Intact	14	21	28
Moment (N*mm)	WT 612.418±236.998	WT 681.582±342.389	WT 470.850±277.999	WT 639.629±344.144
	*Mmp13^−/−^* 539.063±215.845	*Mmp13^−/−^* 610.200±458.343	*Mmp13^−/−^* 735.207±454.051	*Mmp13^−/−^* 512.438±268.329

Results are given as mean ± SD of sample group (intact WT *n* = 10, *Mmp13^−/−^ n* = 10; 14 days post fracture WT *n* = 7, *Mmp13^−/−^ n* = 5; 21 days WT *n* = 2, *Mmp13^−/−^ n* = 7; 28 days WT *n* = 6, *Mmp13^−/−^ n* = 2).

### WT bone marrow-derived cells do not rescue the Mmp13^−/− ^non-stabilized fracture healing phenotype

**Figure 5:**
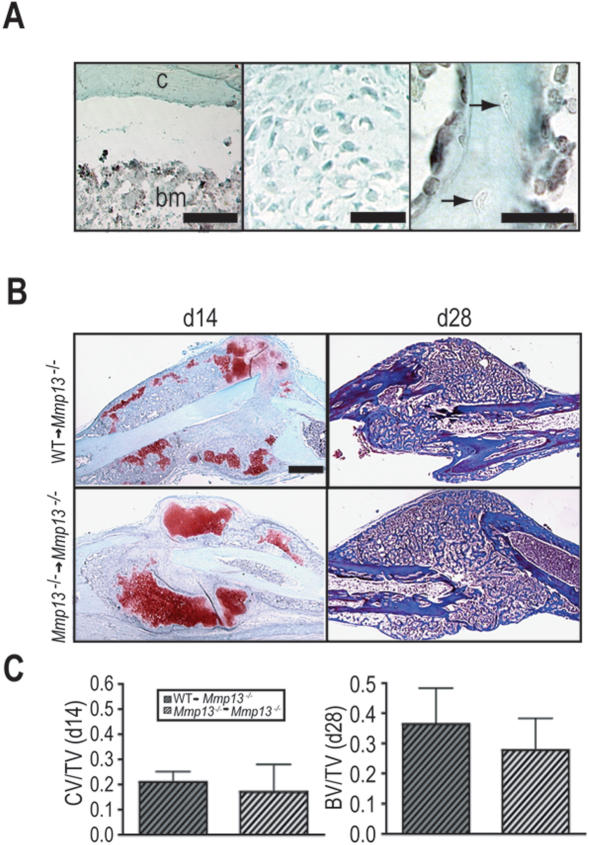
Transplant of WT bone marrow does not rescue the *Mmp13* ^−/− ^non-stabilized fracture healing phenotype. (A) Immunostaining for GFP on callus tissues from *Mmp13^−/−^* mice transplanted with bone marrow from *β-actin GFP* mice (GFP→*Mmp13^−/−^* mice) . (Left panel) Bone marrow cells (bm) are positive for GFP (black staining) showing they are donor-derived but the adjacent cortex (c) is negative. (Middle panel) Chondrocytes at day 14 and (Right panel) osteocytes embedded in the new bone (arrows) at day 28 do not stain for GFP, showing they are host-derived. (B, Left column) SO and (Right column) Masson's Trichrome staining of non-stabilized fracture calluses from *Mmp13* ^−/−^ mice transplanted with WT bone marrow (WT → *Mmp13* ^−/−^) and *Mmp13* ^−/− ^mice transplanted with *Mmp13* ^−/− ^bone marrow (*Mmp13* ^−/− ^→ *Mmp13* ^−/−^) show no difference in the amount of cartilage volume at 14 days post-fracture (WT → *Mmp13^−/−^ n* = 6, *Mmp13^−/−^*→ *Mmp13^−/−^ n* = 5) and no difference in the amount bone at day 28 (WT → *Mmp13^−/−^ n* = 7, *Mmp13^−/−^*→ *Mmp13^−/−^ n* = 4). (C) Histomorphometric analyses of total cartilage volume as a proportion of total callus volume (CV/TV; day 14) and total bone volume as a proportion of total callus volume (BV/TV; day 28) demonstrate no significant difference between WT → *Mmp13* ^−/−^ and *Mmp13* ^−/−^ → *Mmp13* ^−/−^ animals, suggesting that bone marrow transplant does not rescue the *Mmp13* ^−/ ^non-stabilized fracture healing phenotype. Bonferroni corrected t-test, bars represent means ± SD. Scale bars: (A, left and middle) = 50 µm, (A, right) = 25 µm, B = 1 mm.

Osteoclasts are the major bone resorbing cells. Although we did not detect MMP13 in osteoclasts, other cells of hematopoietic origin, specifically in the monocyte/macrophage lineage, can express MMP13 [21]. To distinguish the effects of the *Mmp13* mutation on osteoblasts and chondrocytes from its effects on cells derived from the hematopoietic lineage, we asked whether transplantation of WT bone marrow could rescue the *Mmp13^−/−^* phenotype. As previously observed in WT hosts [22], we confirmed that bone marrow was derived from donor mice (Fig. 5A left panel), while chondrocytes (Fig. 5A, middle panel) and osteoblasts/osteocytes in the callus (Fig. 5A, right panel) were host derived. Histology (Fig. 5B) and histomorphometric analyses (Fig. 5C) showed that there was no difference in the proportion of cartilage in the callus at 14 days post-fracture, or in the proportion of bone in the callus at 28 days post-fracture between *Mmp13^−/−^* mice that received WT (WT→*Mmp13^−/−^*) and *Mmp13^−/−^* (*Mmp13^−/−^*→*Mmp13^−/−^*) bone marrow. These results demonstrate that MMP13-competent hematopoietically derived cells, including osteoclasts are insufficient to restore the timely resorption of cartilage and bone in *Mmp13^−/−^* fracture callus. This suggests that there is an intrinsic defect in chondrocytes, osteoblasts and/or their matrices as the source of the *Mmp13^−/−^* healing phenotype.

### Mmp13 is required for healing by intramembranous ossification

**Figure 6:**
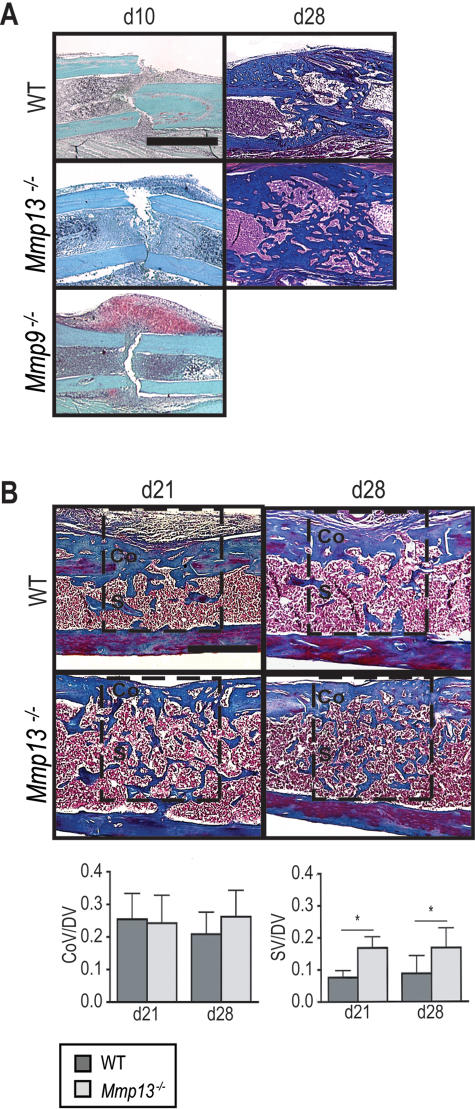
MMP13 is required for normal healing by intramembranous ossification. (A, Left column) SO stain of stabilized fracture calluses at day 10 post-fracture show that unlike *Mmp9* ^−/− ^mice, no cartilage is formed during stabilized fracture healing in *Mmp13* ^−/−^mice (*n* = 14) compared to WT mice (*n* = 3). (A, Right column) At day 28, stabilized fracture calluses in *Mmp13* ^−/−^ mice (*n* = 12) appear to have increased bone volume as compared to WT (*n* = 14) by histology. (B) Masson's Trichrome staining of cortical defect samples at 21 and 28 days post-surgery suggests an increased amount of bone in *Mmp13* ^−/−^mice. Labels designate compact (Co) and spongy (S) regions of defect. Histomorphometric analyses (within the boxed area) of WT (d21 *n* = 5, d28 *n* = 6) and *Mmp13* ^−/−^ (d21 *n* = 6, d28 *n* = 6) cortical defect samples confirm that there is an increase in spongy bone volume (SV/DV) but not compact volume (CoV/DV) in the defect area measured at day 21 and 28 (*p<0.05) in *Mmp13* ^−/−^ as compared to WT. Bonferroni corrected t-test, bars represent means ± SD. Scale bars: A = 1 mm, B = 500 µM.

To differentiate the consequences of the *Mmp13^−/^* ^−^ mutation on cartilage and bone during repair, we examined healing via intramembranous ossification in fractures that were stabilized with a rigid external fixator [4], [5] and in a cortical defect model [23], [24]. WT and *Mmp13^−/−^* mice that received stabilized fractures were examined at 10 and 28 days post-fracture (Fig. 6A). Unlike the *Mmp9^−/−^* mice, which display aberrant cartilage formation at 10 days post-fracture (Fig. 6A bottom left panel; [5]), *Mmp13^−/−^* stabilized fracture calluses (Fig. 6A, middle left panel) did not display cartilage and were comparable to WT (Fig. 6A, top left panel). At 28 days post-fracture, histological analyses suggested that *Mmp13^−/−^* stabilized fracture calluses (Fig. 6A, middle right panel), were larger and contained more bone as compared to WT. However, since differences in callus sizes and bone content may result from variability in the alignment of bone ends at the fracture site, we turned to another model of intramembranous bone healing for quantification.

During healing of cortical defects in long bones, no cartilage is formed [23], [24]. Healing produces new compact bone to repair the damaged compact bone as well as spongy bone in the marrow space underlying the cortical defect. This spongy bone is eventually remodeled and removed [24], [25]. Cortical defects were produced in WT and *Mmp13^−/−^* mice and assessed at 21 or 28 days by histology and histomorphometry. SO staining confirmed that no cartilage was present in the defects at the time points examined regardless of genotype (data not shown). TC staining suggested that there was an increased amount of spongy bone in *Mmp13^−/−^* mice as compared to WT at 21 and 28 days post-injury (Fig. 6B). Histomorphometric analyses of these samples confirmed that while there was no measurable difference in the ratio of compact bone volume as a proportion of total defect volume in these defects, there was a significant increase in the ratio of spongy bone volume as a proportion of defect volume in *Mmp13^−/−^* mice as compared to WT at 21 and 28 days post-surgery (Fig. 6B). These results indicate that the defects observed during late stages of cortical defect healing are similar to the bone remodeling defects observed in non-stabilized fractures. Since cortical defect healing occurs without cartilage intermediates, this model allowed us to produce a bone phenotype in the absence of cartilage perturbations. We conclude that the *Mmp13^−/−^* repair defect occurring in bone can be uncoupled from the defect occurring in cartilage; therefore the *Mmp13^−/−^* mutation overall affected cartilage and bone remodeling independently of one another during fracture repair.

## Discussion

### MMP13 is required for cartilage resorption

We demonstrate here that the absence of MMP13 does not affect the overall amount of cartilage produced in the callus during non-stabilized fracture healing, but rather affects the removal of hypertrophic cartilage from the callus. This is reminiscent of skeletal development in the absence of MMP13, where an accumulation of hypertrophic cartilage results from its delayed removal from the endochondral growth plate [9], [10]. Our study has provided evidence for the importance of MMP13 activity during skeletal repair, which is both complementary to and distinct from its role during development.

**Figure 7:**
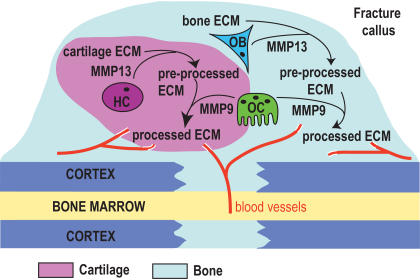
A model for MMP13 action in the cartilage and bone compartments of the non-stabilized fracture callus. MMP13 secreted from hypertrophic chondrocytes (HC) and osteoblasts (OB) acts upon the ECM to produce a pre-processed ECM in both compartments. This pre-processed ECM is then invaded by blood vessels and further modified by osteoclasts (OC) secreting MMP9, leading to the production of a processed ECM. This processed ECM then promotes further steps of callus maturation including hypertrophic chondrocyte apoptosis, replacement of cartilage by bone and new bone remodeling.

Previous studies have also revealed a mechanistic link between fetal skeletal development and adult skeletal repair [3], [5]. The delayed cartilage removal in *Mmp13^−/−^* mice resembles the phenotype that was observed in *Mmp9^−/−^*mice during development and repair of long bones [5], [8]. While MMP9 is strongly expressed by osteoclasts in the fracture callus, we observed that MMP13 was confined to chondrocytes and osteoblasts, suggesting that the *Mmp13^−/−^* and *Mmp9^−/−^* phenotypes may differ at the cellular level. Further analyses of these phenotypes confirmed this assumption. While both angiogenesis and osteoclast recruitment, two events essential for proper and timely resorption of endochondral cartilage during healing are delayed in *Mmp9^−/−^* calluses [5], [8], [15], [26], [27], in contrast, there was no disruption in the recruitment of mature osteoclasts or blood vessels in *Mmp13^−/−^* fracture calluses. However, consistent with our observations, providing a source of WT osteoclasts by bone marrow transplant did not rescue the *Mmp13^−/−^* repair phenotype. This is distinct from the *Mmp9^−/−^* phenotype where the delay in cartilage resorption during fetal bone development was rescued by transplant of WT bone marrow [8]. In addition, the *Mmp9^−/−^* fracture phenotype could be rescued by exogenous VEGF, which stimulated the invasion of the callus by blood vessels and osteoclasts [5]. These results suggest a role for MMP13 prior to vascular invasion and osteoclast recruitment in the process of hypertrophic cartilage remodeling (Fig. 7). The observed decrease in aggrecan cleavage in the absence of MMP13 activity suggests impaired degradation of the cartilage ECM, which slowly resolves over time, in *Mmp13^−/−^* mice. Thus, MMP13 produced by hypertrophic chondrocytes appears to be directly involved in the initiation of hypertrophic cartilage degradation, independent from MMP9 and the recruitment of matrix resorbing cells and blood vessels.

### MMP13 is required in bone remodeling

We found other differences between *Mmp13^−/−^* and *Mmp9^−/−^* phenotypes when we analyzed the production and remodeling of new bone in the fracture callus. In *Mmp9^−/−^* mice, the delayed removal of hypertrophic cartilage correlated with a delay in ossification. On the contrary, *Mmp13^−/−^* mice did not exhibit a delay in the deposition of new bone as a consequence of the cartilage phenotype. Instead, we observed an increase in bone volume at later stages of healing. This increase was restricted to the areas of spongy bone, much like those observed in *Mmp13^−/−^* bones during development [10]. Since spongy bone is lower in density and strength than compact bone, this result may explain why no significant difference was detected when we compared the strength of WT and *Mmp13^−/−^*calluses at late stages of healing.

Examination of stabilized fractures and cortical defects also revealed a role for MMP13 in the timely resorption of the transient compartments of spongy bone during healing via intramembranous ossification. Unlike development, where MMP13 activity is required for the formation of endochondral but not intramembranous bone, these observations suggest that its proteolytic activity is required during repair regardless of the type of ossification involved. These results support the idea that the bone defect observed during adult skeletal repair in the absence of MMP13 is caused by a defect intrinsic to osteoblasts or the matrix they produce. Furthermore, WT osteoclasts did not accelerate the resorption of spongy bone in the *Mmp13^−/−^*callus. Similar to its role in hypertrophic cartilage, MMP13 present in new bone matrix may be required for the initial degradation of ECM components allowing the timely remodeling of the bone callus (Fig. 7).

### Cartilage-bone interaction in the Mmp13^−/−^ fracture callus

That bone remodeling was delayed in both intramembranous and endochondral ossification during bone repair demonstrates that in the absence of MMP13 the cartilage phenotype manifests independently of the bone phenotype. These results are in accordance with the observations reported for skeletal development in the absence of MMP13 by Stickens et al. (2004). In addition, the observed delay in cartilage resorption did not affect the deposition of bone in the *Mmp13^−/−^* callus at day 14 post-fracture. While bone and cartilage have been shown to provide regulatory feedback to each other [28], these results indicate that proper remodeling of the ECM depends on unique signals or properties intrinsic to the bone and cartilage.

Regulatory interactions between bone and cartilage also take place during the early stages of fracture healing [5]. During the early stages of non-stabilized fracture healing in the absence of MMP13, we observed a delay in initial bone deposition correlated with an increase in cartilage formation. This might indicate a transient inhibition of osteogenesis due to increased chondrogenesis or a decreased ability of MMP13-deficient mesenchymal precursor cells to differentiate into fully mature osteoblasts [29]. This early imbalance was not observed during stabilized fracture healing in either WT or *Mmp13^−/−^* mice, which healed via intramembranous ossification. This stands in contrast with *Mmp9^−/−^* mice that heal stabilized fractures via an aberrant endochondral process [5].

### A role for MMP13 in osteoclasts?

The independent manifestation of the cartilage and bone phenotypes may be due to the fact that osteoclasts are not affected in *Mmp13^−/−^* mice. Several studies point to MMPs, possibly even MMP13, as potentially important mediators of osteoclast recruitment during development and repair [30]–[33] and a number of factors that promote bone resorption/remodeling are able to affect *Mmp13* gene expression [34]–[38]. Our results indicate that *Mmp13* is not co-expressed with *Mmp9* in osteoclasts. Furthermore, our bone marrow transplantation studies indicate that WT osteoclasts were not able to support normal ECM remodeling in the *Mmp13^−/−^* environment, suggesting that they did not provide a source of MMP13. This result points again to a defect that is intrinsic to the ECM. MMP13 activity may be required prior to the onset of osteoclastic and MMP9 activity to prepare the bone matrix for resorption (Fig. 7). Indeed, prior work has suggested that MMP13 does participate in the resorption of the organic bone matrix, but that this is through the action of mesenchymal stem cell-derived cells, not osteoclasts [39]. Consequently, WT (and therefore MMP13-competent) osteoclasts are unable to perform their normal function due to a deficiency in this preparatory step in the *Mmp13^−/−^* ECM.

### Implications for normal skeletal repair

In humans, although the mode of healing may vary depending on the site of injury, extent of trauma, and fixation, most fractures heal through endochondral ossification. Therefore in the majority of clinical cases, remodeling *o*f both cartilage and bone is critical for bone bridging and full recovery of mechanical integrity. While most fractures heal spontaneously, 5 to 10% fail to heal in a timely manner [40]. Risk factors such as age, injury type and socio-economic conditions have been shown to impact the outcome of healing, yet little is known about the genetic predispositions to impaired healing. Although mutations in MMP genes have been linked to skeletal defects in humans [12], [41], none have been associated with delayed healing. Our results show that MMP13 and other MMPs such as MMP9 play a role in all stages of fracture healing from the early deposition of cartilage and bone to the late stages of callus remodeling. Perturbations in any of these processes may compromise healing. Although *Mmp13^−/−^* mice have phenotypic manifestations similar to *Mmp9^−/−^* mutants, such as late onset of hypertrophic cartilage removal, we show that there are intrinsic differences in the way these two proteases act in fracture repair and that treatment strategies to overcome these defects differ. These observations point to the need for a better understanding of the underlying causes of delayed healing in humans in order to plan efficient therapies. From this perspective, the detailed analyses of *Mmp9^−/−^* and *Mmp13^−/−^*mutant mice shed light into the normal process of bone healing and potential approaches to treat impaired healing.

## Materials and Methods

### Non-stabilized and stabilized fractures

*Mmp13* ^−/−^ mice (3- to 6-month-old males; 30–35 grams (g)) and their WT littermates were anesthetized with an intraperitoneal injection of 50 mg/ml Ketamine/0.5 mg/ml Medetomidine (0.03 ml/mouse). Closed, standardized non-stabilized fractures were produced as previously described [5]. For stabilized fractures, an external fixator was placed as previously described [4]. Mice were sacrificed by cervical dislocation following an intraperitoneal injection of 2% Avertin (0.5 ml/mouse). Non-stabilized fractures were collected at 5, 10, 14, 21, 28, and 56 days post-fracture. Stabilized fractures were collected at 10 and 28 days post-fracture. All protocols were approved by the Institutional Animal Care and Use Committee at UCSF.

### Bone marrow transplantation

10-week-old male *Mmp13^−/−^* mice were lethally irradiated with two 6 Gy doses of γ-irradiation 3–4 hours apart. Bone marrow cells from WT, *Mmp13^−/−^* and *β-actin Green Fluorescent Protein* (*GFP*) mice were transplanted into irradiated *Mmp13^−/−^* mice from the same FVB/N background as previously described (Colnot et al., 2006). Following a recovery period (Colnot et al. 2006), non-stabilized fractures were produced in chimeric mice as described above. Fracture tissues were collected at 14 and 28 days post-fracture and processed for histological analyses.

### Cortical defects

Monocortical defects (1mm in diameter) were produced on the anterior-proximal tibia as previously described [23], [24]. Samples were collected at 21 and 28 days post-surgery and processed for paraffin embedding. Longitudinal sections parallel to the plane of the defect were collected and processed for histological analyses.

### Histology and immunohistochemistry

Callus tissues were fixed overnight at 4°C in 4% paraformaldehyde, decalcified at 4°C in 19% EDTA (pH 7.4) for 10–14 days, then dehydrated in a graded ethanol series and embedded in paraffin. Sections (10 µm thick) were stained with Safranin-O/Fast Green (SO) to detect cartilage formation as described [4]. A modified Milligan's Trichrome (TC) staining using Analine Blue was performed to analyze bone formation in the fracture callus. Tartrate-resistant acid phosphatase (TRAP) staining was performed using a leukocyte acid phosphatase kit (Sigma, St. Louis, MO) as previously described [16]. Immunohistochemistry for platelet endothelial cell adhesion molecule-1 (PECAM) was done as previously described [5], [8]. Immunohistochemistry for the DIPEN epitope was performed as described previously [10].

Calluses collected from *Mmp13^−/−^* mice transplanted with bone marrow from *β-actin GFP* mice were fixed and decalcified as mentioned above, then cryo-embedded in OCT. Sections (8 µm) were cut on a cryostat (Leica). For GFP immunostaining, cryosections were treated with 0.3%H_2_O_2_ in methanol, digested with ficin (Zymed), and blocked with 5% milk in PBS and 5% normal goat serum in PBS. Antibody staining was done using rabbit anti-GFP antibody (Abcam) followed by goat anti-rabbit secondary antibody conjugated to horseradish peroxidase. Slides were developed with diaminobenzidine and counterstained with 0.1% fast green.

### In situ hybridization

In situ hybridization was performed using ^35^S-labeled probes from mouse cDNAs for *Mmp13*, *Col1* α1 chain (*Col1a1* – Mouse Genome Informatics), *Mmp9*, *Col10* (*Col10a1* – Mouse Genome Informatics), *Vegf* (*Vegfa* – Mouse Genome Informatics), *Oc* (*Tcirg1* – Mouse Genome Informatics) and *Col2* (*Col2a1* – Mouse Genome Informatics) as previously described [16]. Emulsion coating and image analyses were performed as described previously [2], [42].

### Histomorphometric measurements

Histomorphometry on fracture samples was performed as previously described [5], [43]. To determine the amount of cartilage within each callus, every thirtieth section (300 µm) was stained with SO. Adobe Photohsop was used to capture images from a Leica DM 5000 B light microscope (Leica Microsystems GmbH, Wetzler, Germany) that was equipped with a camera (Diagnostic Instruments, Inc., Sterling Heights, MI). To determine the amount of bone within each callus, adjacent sections were stained with TC and photographed. The area of the callus, cartilage and bone (including compact and spongy bone [44] was determined using Adobe PhotoShop.

To determine the amount of bone within cortical defects, 2 central sections within the defect were selected and stained with TC. In Adobe PhotoShop, a standard box (as shown in Figure 6) was used to select an area in the center of the defect, including the damaged cortical region and the underlying bone marrow space. The total area of bone within the defect, as well as the areas of compact and spongy bone (Steele, 1988) within each standard box were quantified and the ratios of compact repair/defect volume and spongy repair/defect volume were calculated.

### Mechanical testing

Mechanical testing was performed on intact tibiae from 12-week-old mice and tibiae from mice that had received non-stabilized fractures collected at 14, 21 and 28 days post-fracture. The proximal and distal end of each tibia was potted in a cryovial cap (Corning Incorporated Life Sciences, Lowell, MA) in polymethylmethacrylate (PMMA) bone cement at room temperature. Potted samples were stored overnight at 4°C until testing [45]. Prior to testing, samples were warmed to room temperature for at least 30 minutes; any intact fibulae were removed and mineral-free PBS was applied to the calluses after mounting into the testing apparatus. Tests were performed at room temperature. Data was collected using LabView4 (National Instruments Corporation, Austin, TX).

Each potted mouse tibia specimen was placed horizontally into a uniaxial test frame to prevent gravitational loading of the specimen by the PMMA endcaps [46]. The distal end of the specimen was rigidly held to the test frame while the proximal end was attached to a single-cable pulley system. Movement of the test frame actuator caused tension in the cable, which was positioned around a loading ring so as to generate a pure moment, or couple, loading condition. A uniaxial load cell (Sensotec Model 129, 25 lb capacity, Honeywell International, Inc., Columbus, OH) was used to monitor cable tension, and the moment applied to the specimen was calculated as the product of the cable tension and the diameter of the loading ring (N*mm). Moment and crosshead displacement were monitored, and the ultimate moment over the entire destructive test cycle was recorded as the failure strength of the specimen.

### Statistical analyses

For non-stabilized fracture studies, Wilcoxon rank sum tests were used at each timepoint to examine whether differences between WT and *Mmp13^−/−^* samples in cartilage, callus, and bone volumes were statistically significant (α<0.05). Bonferroni adjustments were performed. For mechanical testing of non-stabilized fracture calluses, force data were analyzed using GraphPad Prism 4 by one-way analysis of variance (ANOVA). For bone marrow transplant and cortical defect studies, histomorphometry data were analyzed using ANOVA followed by Bonferroni corrected t-tests for data sets where ANOVA, p<0.05.
